# Novel Biological Activities of Allosamidins

**DOI:** 10.3390/molecules18066952

**Published:** 2013-06-13

**Authors:** Shohei Sakuda, Hiromasa Inoue, Hiromichi Nagasawa

**Affiliations:** 1 Department of Applied Biological Chemistry, the University of Tokyo, Bunkyo-ku, Tokyo 113-8657, Japan; 2 Department of Pulmonary Medicine, Kagoshima University, 8-35-1 Sakuragaoka, Kagoshima 890-8520, Japan

**Keywords:** allosamidin, chitinase, inhibitor, asthma, *Streptomyces*, secondary metabolite

## Abstract

Allosamidins, which are secondary metabolites of the *Streptomyces* species, have chitin-mimic pseudotrisaccharide structures. They bind to catalytic centers of all family 18 chitinases and inhibit their enzymatic activity. Allosamidins have been used as chitinase inhibitors to investigate the physiological roles of chitinases in a variety of organisms. Two prominent biological activities of allosamidins were discovered, where one has anti-asthmatic activity in mammals, while the other has the chitinase-production- promoting activity in allosamidin-producing S*treptomyces*. In this article, recent studies on the novel biological activities of allosamidins are reviewed.

## 1. Introduction

Chitin, a polymer of β-1,4 linked *N*-acetyl-D-glucosamine, is an important biomass, second only to cellulose in abundance in Nature. Its occurrence in living organisms is specialized, such as in insect cuticles, fungal cell walls, and crab shells [1]. In these chitin-containing organisms, chitin is a polysaccharide constituent that is essential for their growth, therefore, chitin is an ideal target for developing insecticides or fungicides with high selectivity [2,3]. Inhibitors of enzymes responsible for chitin metabolism in the chitin-containing organisms are possible candidates for useful drugs. Chitin synthase and chitinase are key enzymes for chitin synthesis and degradation, respectively. Chitin synthase is present only in chitin-containing organisms and is critical for their growth. Therefore, inhibitors of this enzyme such as polyoxins and nikkomycins are used practically as high selectivity insecticides or fungicides [4]. On the other hand, chitinases are ubiquitously present not only in chitin-containing organisms, but also in non-chitin-containing organisms such as bacteria, plants, or mammals. Chitinases have a variety of physiological roles, and therefore basic studies on the specific chitinase present in each organism are very important for understanding these properties and physiological roles. Specific chitinase inhibitors are useful not only as potential insecticide or fungicide candidates, but also as probes for basic research [5].

**Figure 1:**
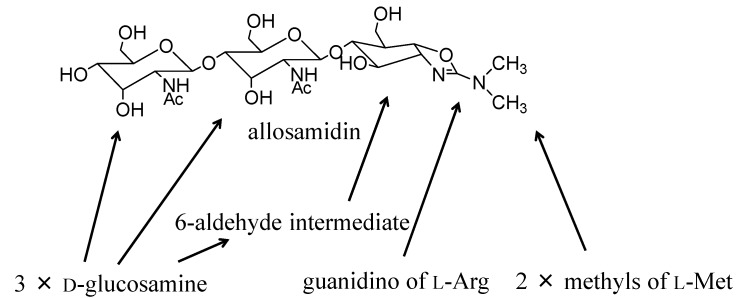
Structure and biosynthesis of allosamidin.

**Figure 2:**
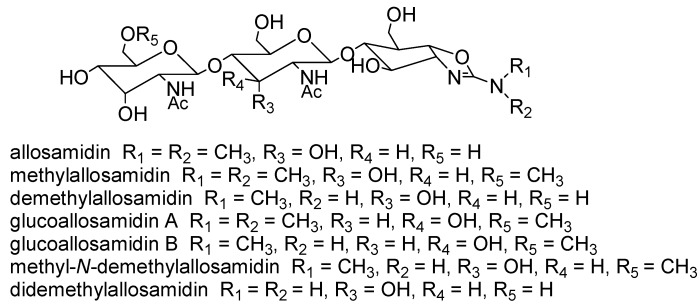
Structures of natural allosamidins.

Allosamidin (Figure 1), a metabolite of a soil bacterium *Streptomyces* sp., was discovered in 1986 as the first chitinase inhibitor [6,7]. It has a unique pseudotrisaccharide structure consisting of two *N*-acetyl-D-allosamine moieties and one allosamizoline moiety [8,9]. Allosamizoline is a unique five-membered cyclitol derivative fused with a dimethylaminooxazoline ring. Biosynthesis of allosamidin was studied using incorporation experiments with a variety of labeled precursors (Figure 1) [10,11]. Both allosamine and the 2-aminocyclitol skeleton originate from the D-glucosamine molecule, and the (CH_3_)_2_N-C moiety of allosamizoline comes from a methyl group of L-methionine and a guanidino group of L-arginine. The cyclopentane ring of allosamizoline was shown to be likely formed through a 6-aldehyde intermediate [12]. To date, seven natural allosamidins have been isolated (Figure 2) [13]. The chemistry of allosamidin and derivatives, including synthetic and X-ray crystallographic studies, have been reviewed by many researchers [14,15,16].

**Scheme 1:**
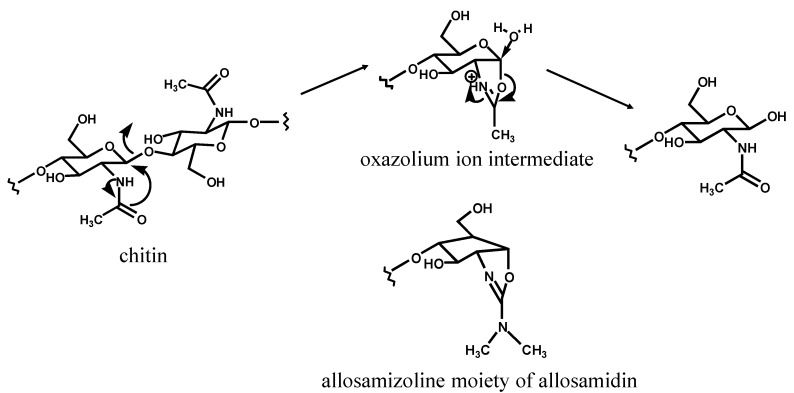
Mechanism of inhibition of family 18 chitinase by allosamidin.

Allosamidin can inhibit all family 18 chitinases, but does not inhibit family 19 chitinases [17,18,19]. Family 18 chitinases cleave chitin to yield the β configuration at C1 by a mechanism that leads to retention of the anomeric configuration after hydrolysis. On the other hand, the reaction mediated by family 19 chitinases yields the α configuration at C1 with an inversion of the anomeric configuration. Family 18 chitinases are believed to catalyze chitin hydrolysis through a substrate-assisted mechanism in which an oxazolium ion intermediate is produced during the enzymatic reaction [20]. The allosamizoline moiety of allosamidin may bind to the active center as a mimic of the intermediate [21], leading to inhibition of the enzyme reaction (Scheme 1). Allosamidin binding with family 18 chitinases have been further examined by theoretical [22], NMR [23], X-ray crystallographic [24,25,26,27,28], and thermodynamic [29,30,31] studies.

Allosamidins have been used to investigate physiological roles in a variety of organisms, which are summarized in Table 1. Allosamidins inhibit insect moulting [7,32,33,34] and the cell separation of yeast [35,36], indicating that these enzymes play an essential role in degrading the old cuticle and during fission of the septum, respectively. In fungi, it has been postulated that chitinase is necessary for fungal growth such as hyphal growth and branching [37], but a critical effect of allosamidins in fungal growth has not been observed [38,39], though a weak fungistatic effect of allosamidin on autolyzing *Penicillium* mycelia has been reported [40]. Therefore, it is still unclear if a chitinase inhibitor would be a potentially efficacious antifungal agent. Allosamidin inhibits encystment of a parasite [41] and the transmission of the malaria parasite in mosquito models [42,43,44,45], indicating that chitinases present in malaria ookinetes play a role for the penetration of ookinetes into the midgut epithelium of the mosquito by degrading the peritrophic matrix. In plants, one of the roles of chitinases may be a defensive function. It was found that allosamidin enhanced stress tolerance of plants [46], but molecular mechanism of allosamidin’s effects on plants is unknown. Bacteria may produce chitinases to obtain nutrients through degradation of environmental chitin and to invade into host fungi [47,48]. We have shown the physiological role of allosamidin in its producing *Streptomyces*. Allosamidin promotes chitinase production and growth of its producer [49,50,51]. In mammals, the physiological role of chitinases remains unclear. It was reported that allosamidin shows anti-asthmatic activity in a mouse model of asthma [52] and reduces inflammatory signs observed in endotoxin-induced uveitis in rabbits [53]. In addition, it was reported that allosamidin promotes atherosclerosis in hyperlipidemic mice [54].

**Table 1:** Roles of chitinases and allosamidin’s effects

Organisms	Roles of Chitinases	Allosamidin’s Effects	Reference
**insects**	degradation of old culticle	inhibition of moulting	[7,32,33,34]
**yeasts**	fission of septum	inhibition of cell separation	[35,36]
**fungi**	(hyphal growth and branching) ^a^	no significant effect	
**parasites**	degradation of peritrophic matrix	inhibition of transmission of	[42,43,44,45]
		malaria ookinetes	
		inhibition of encystment	[41]
**plants**	(defensive function) ^a^	enhancement of stress tolerance	[46]
**bacteria**	obtaining nutrient	promotion of chitinase production of *Streptomyces*	[49,50,51]
**mammals**	(defensive function) ^a^	anti-asthmatic activity	[52,68]

^a^ Parentheses mean speculative roles.

In this article, recent works on these chitinase-production promoting and anti-asthmatic activities of allosamidins are reviewed.

## 2. Physiological Roles of Allosamidin in its Producing *Streptomyces*

### 2.1. Microbial Secondary Metabolites 

Microorganisms can produce a variety of secondary metabolites, which have been used as important sources for developing useful drugs, including medicines, pesticides and perfumes. The novelty of the structures and biological activities of microbial secondary metabolites are of great interest in the basic sciences. Genome analyses have clarified that a strain of a microbe, such as *Streptomyces* sp. or *Aspergillus* sp., has the ability to produce dozens of secondary metabolites. Recent extensive information on biosynthesis of microbial secondary metabolites indicates that the substrate specificity of biosynthetic enzymes involved in secondary metabolism is high. These facts suggest that microorganisms have developed secondary metabolism mechanisms during evolution and that each secondary metabolite might have a physiological role in respective organism under certain circumstances. The role of antibiotic production is presumable but has not been proved. It is entirely unknown why microorganisms produce numerous other compounds without antibiotic activity, such as enzyme inhibitors.

### 2.2. Streptomyces Chitinases

Soil is rich in chitin originated from chitin-containing soil organisms, such as fungi and insects. Therefore, chitinases are important for soil bacteria in order to obtain chitin as a nutrient source. *Streptomyces* is thought to be a main microbe for the degradation of chitin in soil. A medium containing chitin as a sole carbon source can be used for the rough selection of *Streptomyces* [55]. There are many chitinase genes in a *Streptomyces* genome. For example, six family 18 chitinase genes and two family 19 chitinase genes are present in the *S. coelicolor* genome [56]. However, the regulatory mechanism of chitinase expression in *Streptomyces* has not been fully clarified yet. It is known that glucose suppresses the expression of chitinase genes with a common direct repeat sequence in the promoter region of the genes [57], and that *N*,*N’*-diacetylchitobiose [(GlcNAc)_2_] induces chitinase expression by releasing the suppression [58]. These facts strongly suggest the presence of a suppressor protein that may bind to the direct repeat sequence in the presence of glucose and detach from it in the presence of (GlcNAc)_2_, but the putative suppressor protein has not been identified. (GlcNAc)_2_ is the main product of chitinase-mediated processing of chitin and a specific transporter for (GlcNAc)_2_ has been identified in *Streptomyces* [59].

### 2.3. Chitinase Production Promoting Activity of Allosamidin in an Allosamidin-Producing Strain

**Figure 3:**
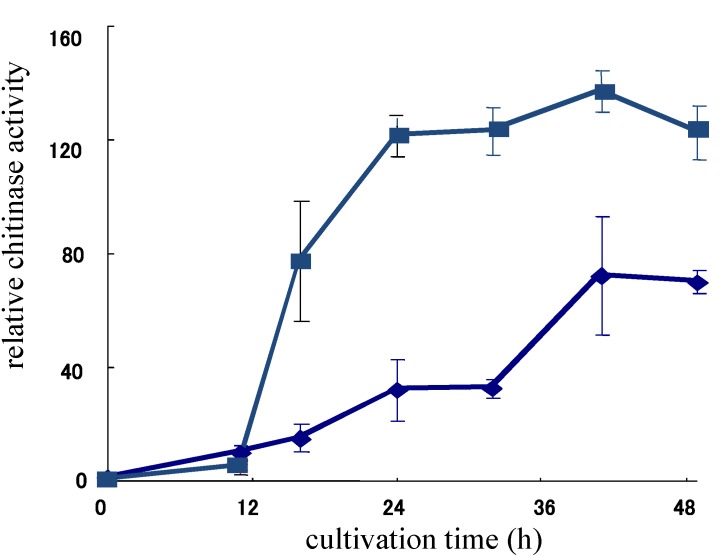
Chitinase production of *Streptomyces* sp. AJ9463 with (■) or without (◆) allosamidin (2 μM) in colloidal chitin medium.

Allosamidin is a typical secondary metabolite of *Streptomyces* sp. and allosamidin-producing strains are easily obtained from soil. Approximately 5% of randomly isolated *Streptomyces* strains produce allosamidin, suggesting that allosamidin might play a role in soil. To obtain a clue for investigating the physiological role of allosamidin, we first focused on the allosamidin-insensitive chitinase produced by an allosamidin-producing strain, *Streptomyces* sp. AJ9463, because no family 19 chitinase had been found in any bacteria other than strain AJ9463 at that time [60]. However, it was shown that *Streptomyces griseus* produced a family 19 chitinase [61] and an allosamidin-insensitive chitinase was not specially produced by allosamidin producers [62], but during the work for optimizing a medium suitable for chitinase production by strain AJ9463, we found that the addition of allosamidin into a chitin medium could enhance chitinase production of strain AJ9463 (Figure 3) [63]. This finding prompted us to elucidate the molecular mechanism of allosamidin’s effect on chitinase production.

Allosamidin enhanced chitinase activity of the culture filtrate of strain AJ9463 in a colloidal chitin medium at a concentration of 0.06–2.0 μM in a dose-depending manner [49]. Analysis of the chitinases in the culture filtrate by activity staining showed that two predominant chitinases (46 kDa and 105 kDa) were produced and that the amounts of both chitinases increased by addition of allosamidin in a dose-dependent manner. Since both chitinases were produced in the medium without allosamidin, chitinase production appeared to be promoted by allosamidin. The enzymatic activity of the chitinases was inhibited by allosamidin at a concentration of more than 10 μM, but inhibition was not observed at the concentration of 0.06–2.0 μM. Furthermore, the amount of allosamidin produced by allosamidin producers was less than 1.0 μM in the culture broth. These facts suggest that allosamidin is produced at a physiologically significant concentration.

Chitinase production may strongly affect growth when a bacterium is grown in a medium containing chitin as a sole carbon source. The addition of allosamidin clearly promoted mycelial growth in strain AJ9463, suggesting that allosamidin’s effect on chitinase production may be physiologically important in its producer.

### 2.4. Molecular Mechanism of Allosamidin’s Function on Chitinase Production

**Figure 4:**
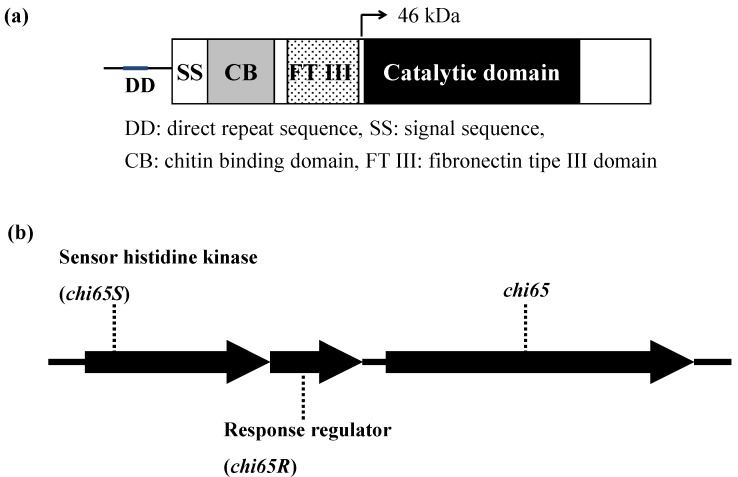
Schematic representation of *chi65* (**a**), *chi65R* and *chi65S* (**b**).

The 46 kDa chitinase, whose production is enhanced by allosamidin, was found to be encoded by the *chi65* gene. The amino acid sequence of Chi65 protein deduced from the nucleotide sequence of the gene showed that it contained chitin binding domain, fibronectin type III domain, and catalytic domain, but 46 kDa chitinase lacked the chitin binding and fibronectin type III domains (Figure 4) [50]. The 105 kDa chitinase was thought to be a dimer of the 46 kDa protein because the two chitinases had the same amino acid sequence [49]. A direct repeat sequence was present in the promoter region of *chi65*, and two genes (*chi65S* and *chi65R*) encoding a sensor histidine kinase and response regulator, respectively, were present at the 5'-upstream region of *chi65* (Figure 4) and can comprise a two-component regulatory system. This suggested that expression of Chi65 was regulated not only by the mechanism related to the direct repeat sequence and suppressor protein, but also by the regulation concerning a two-component system of Chi65S and Chi65R.

(GlcNAc)_2_ is a key regulator for chitinase production in *Streptomyces* as described above. (GlcNAc)_2_ enhanced production of 46 kDa and 105 kDa chitinases under the same conditions that were used to test the effect of as allosamidin, but its activity was much weaker than that of allosamidin. A mutant strain with disrupted *chi65R* and *chi65S* genes was found to be sensitive to (GlcNAc)_2_, but it became insensitive to allosamidin. This results indicated that (GlcNAc)_2_, but not allosamidin, can act on the putative suppressor and that allosamidin enhances Chi65 production through the two-component regulatory system. In the experiments with a medium containing only inorganic salts, allosamidin did not promote chitinase production but (GlcNAc)_2_ did induce production of 46 kDa and 105 kDa chitinases. Furthermore, allosamidin enhanced chitinase production under the presence of (GlcNAc)_2_ in the inorganic salts medium, indicating that (GlcNAc)_2_ is necessary for the function of allosamidin. These findings suggested the following regulatory mechanism for Chi65 expression. First, (GlcNAc)_2_ binds to the suppressor and expression of *chi65* starts. Allosamidin may subsequently act on the sensor region of Chi65S and strongly enhance *chi65* expression through the two-component regulatory system.

### 2.5. Localization of Allosamidin

Allosamidin localizes in the mycelia of strain AJ9463 when it is cultured in a medium without chitin. This localization creates a big problem if allosamidin is supposed to act on a membrane sensor from outside of the cells. However, we could find a phenomenon that allosamidin is released into a culture filtrate from the mycelia in a chitin medium. Chitin or its degradation product was hypothesized to act as a releasing factor during the process of allosamidin release, and the inducing factor was identified as (GlcNAc)_2_ through the experiments with the inorganic salts medium (Suzuki *et al.*, unpublished data). (GlcNAc)_2_ was found to induce allosamidin release from the strain AJ9463 cells at a concentration of several micromolar, which is a similar concentration that induced chitinase production. Therefore, it was speculated that chitinase production and allosamidin release were induced by (GlcNAc)_2_ in a successive manner.

### 2.6. Generality of the Action of Allosamidin in Streptomyces

Allosamidin enhances production of the chitinase originated from *chi65h* of *Streptomyces halstedii* MF425, which is an allosamidin producer [51]. *Chi65h* is highly homologous to *chi65*, and the direct repeat sequence is present in the promoter region of *chi65h* and two genes homologous to *chi65S* and *chi65R* were present at the 5'-upstream region of *chi65h*. Allosamidin also enhanced chitinase production of allosamidin non-producers, *Streptomyces coelicolor* A3(2) and *Streptomyces griseus*. It promoted production of chitinases encoded by *chiC* of *S. coelicolor* A3(2) and *chiIII* of *S. griseus*, which have high homology to *chi65*. Two genes homologous to *chi65S* and *chi65R* were also present at their 5'-upstream regions. Furthermore, when allosamidin’s effect was tested with six *Streptomyces* strains randomly isolated from soil, allosamidin enhanced chitinase production in all of the strains. All six strains possessed a set of three genes homologous to *chi65*, *chi65S*, and *chi65R*. Analysis of 16S rDNA indicated that allosamidin-sensitive strains are widely distributed in *Streptomyces*. These observations suggest that allosamidin can affect the common regulatory system for production of a chitinase with a two-component regulatory system in *Streptomyces*.

### 2.7. Role of Allosamidin in Nature

**Scheme 2:**
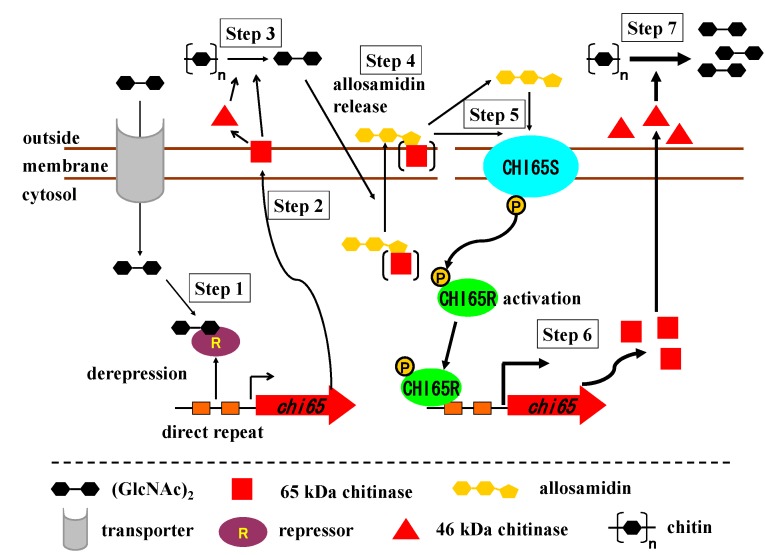
Putative molecular mechanism of allosamidin’s function on chitinase production in allosamidin-producing *Streptomyces.*

The 46 kDa chitinase was observed in the culture filtrate of strain AJ9463 when cultured in a chitin medium. The *chi65* gene was speculated to produce 65 kDa chitinase. The 65 kDa protein was not present in the culture filtrate, but was detectable in the membrane fraction of strain AJ9463. Based on these observations, we propose the following model of the function of allosamidin (Scheme 2). When strain AJ9463 present in soil comes in contact with chitin, a small amount of (GlcNAc)_2_ is produced by the action of chitinase of the strain itself or other microbes, and it induces Chi65 production by binding to the suppressor (Scheme 2, step 1). Chi65 is secreted from the cells, but still located on the membrane as the 65 kDa protein (step 2). After proteolysis of the 65 kDa protein, 46 kDa chitinase is produced, which is secreted out of cells. The concentration of (GlcNAc)_2_ produced by degradation of chitin increases rapidly by the action of the 65 kDa and 45 kDa chitinases (step 3). Allosamidin is transferred to outside of the cells by attaching Chi65 or by an unknown mechanism induced by (GlcNAc)_2_ produced (step 4). Allosamidin or allosamidin-chitinase complex binds to the sensor moiety of Chi65S (step 5), leading to enhancement of Chi65 expression using the two-component regulatory system of Chi65S and Chi65R (step 6). The concentration of (GlcNAc)_2_ further increases dramatically by the action of the 65 kDa and 45 kDa chitinases as well as other chitinases whose expression are induced by (GlcNAc)_2_ (step 7). Since (GlcNAc)_2_ is used as a nutrient, the growth of the bacterium is dramatically promoted. Allosamidin also affects the production and activity of chitinases of neighboring bacteria. It is hypothesized that allosamidin can enhance chitinase production of almost all *Streptomyces* strains and could inhibit the chitinase activities of other bacteria, leading to nutrient conditions suitable for the growth of *Streptomyces*. In our preliminary model experiment, colonies of *Streptomyces* clearly increased when soil was cultured in a chitin medium containing allosamidin. These findings suggest that allosamidin may act as an important signal molecule for chitin metabolism in an environment such as soil.

## 3. Anti-Asthmatic Activity of Allosamidins

### 3.1. Asthma and Acidic Mammalian Chitinase

**Figure 5:**
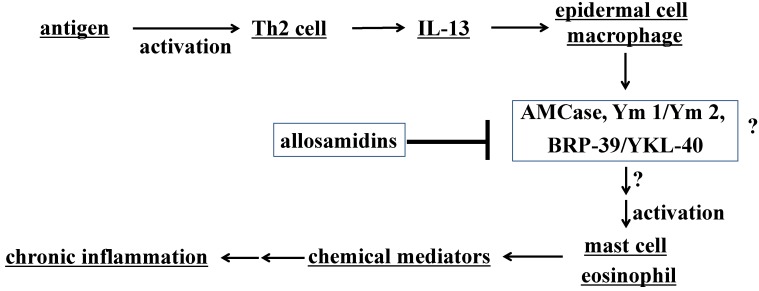
Asthma, and chitinase and chitinase-like proteins.

Bronchial asthma is a chronic inflammatory disease characterized by eosinophilic infiltration, airway hyperresponsiveness to non-specific stimuli, and remodeling of the airways. Today, asthma prevalence has reached 5% worldwide, and therefore more effective drugs are necessary to cure the disease, especially intractable asthma. T-helper-2 (Th2) cytokines are essential for generating asthmatic abnormalities. Among Th2 cytokines, IL-13 is now considered particularly critical (Figure 5) [64].

Although chitin is not present in mammals, two chitinases, chitotriosidase and acidic mammalian chitinase (AMCase), are present in human and mouse [65,66]. The physiological roles of the chitinases are not clear, but it is speculated that one of their roles is a defensive function against pathogens. AMCase has recently been associated with animal models of asthma. It was reported that AMCase expression is upregulated in the response to allergen exposure or IL-13-induced inflammation in the lung [52,67]. Inhibition of AMCase with anti-acidic mammalian chitinase sera leads to lower eosinophil counts and reduction in airway hyper-responsiveness in a murine model of asthma. Allosamidin was found to suppress allergen-induced airway eosinophilia in the asthma model [52] and inhibition of AMCase by allosamidin was reported [65]. Therefore, the effect of allosamidin on asthma may support the importance of AMCase in the mouse asthma. These observations suggest that AMCase acts as a proinflammatory mediator in IL-13 effector responses (Figure 5), and thus a compound with stronger AMCase inhibitory activity would be expected to show stronger anti-asthmatic activity than allosamidin.

Demethylallosamidin is an allosamidin congener with a monomethylamino group (Figure 2) and has much stronger inhibitory activity toward yeast chitinases [35] and human chitotriosidase [27]. AMCase and chitotriosidase have more than 50% amino acid sequence similarity in both human and mouse enzymes. Therefore, we expected that demethylallosamidin inhibits AMCase more strongly than allosamidin and shows stronger anti-asthmatic activity.

### 3.2. Activities of Allosamidin and Demethylallosamidin on AMCase and Asthma

Recombinant mouse-AMCase expressed in COS-7 cells was used to test the inhibitory activity of allosamidins and demethylallosamidin on AMCase. The inhibitory activity of the two compounds did not differ in the pH range of 2 to 7.5 [68]. Both compounds inhibited AMCase more strongly at neutral pH than an acidic pH, similarly to the cases observed in other chitinases [69].

In contrast, the two compounds exhibited very different *in vivo* activities in the IL-13-induced asthmatic model of mice. IL-13 and allosamidin or demethylallosamidin were administered to mice intratracheally and intraperitoneally, respectively. IL-13 treatment induced eosinophilia counts and eotaxin concentration in bronchoalveolar lavage (BAL) fluid. Allosamidin or demethylallosamidin decreased the eosinophil counts and eotaxin concentration in a dose-depending manner. Very interestingly, demethylallosamidin was much more effective than allosamidin. Allosamidin and demethylallosamidin completely inhibited IL-13-induced eosinophilia and eotaxin at 10 and 1 mg/kg, respectively. IL-13 also induced airway hyperresposiveness to inhaled acetylcholine. Airway pressure was increased by administration of IL-13. 10 mg/kg allosamidin did not decrease the airway pressure to the control level, but 1 mg/kg demethylallosamidin did it. These results indicate only demethylallosamidin can suppress IL-13-induced hyperresponsiveness and has a much superior potential than allosamidin as an anti-asthmatic agent [68].

IL-13 enhanced the chitinase activity and AMC expression in BAL fluid. The AMCase expression enhanced by IL-13 was not inhibited by allosamidin or demethylallosamdin. The chitinase activity in BAL fluid increased by IL-13 was decreased by 10 mg/kg allosamidin or 1 mg/kg demethylallosamidin. The decreased levels of chitinase activity coincided with the amount of inhibitors, but did not coincide with the inhibitory activity on eosinophil counts or airway hyperresponsiveness of the two compounds. These results suggest that it may be necessary to consider other target molecules to explain the difference between anti-asthmatic activities of allosamidin and demethylallosamidin.

### 3.3. Targets of Allosamidins for Their Anti-asthmatic Activity

**Figure 6:**
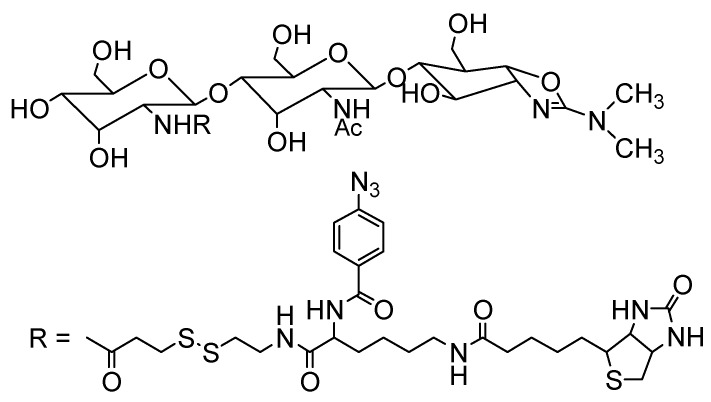
Photoaffinity probe of allosamidin.

Photoaffinity probes of allosamidin and demethylallosamidin were prepared to investigate their binding proteins (Figure 6) [70]. The photoaffinity probes possess photoactive aryl azido moiety and biotin moiety. They maintain strong inhibitory activities toward *Trichoderma* chitinase. By the experiments with these probes, Ym1 was identified as a possible allosamidin-binding protein present in the BAL fluid of IL-13 induced asthmatic mice. Ym1 belongs to chitinase-like proteins that have structures homologous to chitinases but do not have chitinase activity due to a lack of essential amino acid residue(s) commonly present at the active site of family 18 chitinases [71,72]. Chitinase-like proteins are present in a wide range of organisms including mammals, insects, and plants. However, little is known about their physiological roles at the molecular level. The chitinase-like proteins in mouse include Ym 1, Ym 2 (Ym 1 homolog), BRP-39, and oviductin, and those in human include YKL-40 (human homolog of BRP-39), YKL-39, and oviductin. Some of them can bind to poly and/or oligosaccharide. Ym 1 has been reported to bind to chitin [73], and YKL-40 and YKL-39 can bind to *N*-acetylglucosamine oligomers [74,75]. The carbohydrate-binding properties of chitinase-like proteins are thought to be involved in their biological activity. Therefore, allosamidins may have the potential to inhibit the function of these proteins by binding to them.

It has been suggested that Ym 1 and BRP-39/YKL-40 have an important role in asthma, similarly to AMCase [71,72,76]. The expression of Ym 1 and BRP-39 was upregulated in the lung of mouse asthma model [77,78]. Moreover, Ym 1 induced eosinophil chemotaxis and anti-sense Ym1 RNA suppressed asthmatic responses in the model [77]. It was suggested that Ym1/Ym 2 inhibited 12/15(*S*)-lipoxygenase and promoted Th2 cytokine production [79]. The knockout of BRP-39 attenuated asthmatic responses in mice [78], and thus BRP-39 may function as an effector molecule of IL-13. BRP-39 and AMCase were shown to suppress apoptosis and a mutant AMCase lacking chitinase activity also showed apoptosis suppression activity [79]. A strong correlation between YKL-40 expression levels in lung and human asthma has been demonstrated [78]. These reports may suggest that all of Ym 1, Ym 2, BRP-39, and AMCase have a potential as targets of allosamidins in mice (Figure 5). Further studies are needed to investigate the action of allosamidin and demethylallosamidin on each of these proteins in detail to “understand why demethylallosamidin shows much stronger anti-asthmatic activity than allosamidin”.

## 4. Conclusions 

Allosamidin has been used as a probe in basic research for studying chitinases since it was first discovered in 1986. The finding of the chitinase-production promoting activity of allosamidin may provide a new angle for the field of research studying microbial secondary metabolites. Studies on the physiological roles of secondary metabolites may be very important not only as basic researches but also as their application to agriculture kind for environment. The anti-asthmatic activity of allosamidins indicates their potential used not only as lead compounds for developing effective anti-asthmatic drugs but also as probes for investigating physiological roles of chitinase-like proteins.

## References

[1] Muzzarelli R.A.A. (1977). Chitin.

[2] Kramer K.J., Koga D. (1986). Insect chitin; physical state, synthesis, degradation and metabolic regulation. Insect biochem..

[3] Cohen E. (1987). Chitin biochemistry; Synthesis and inhibition. Ann. Rev. Entomol..

[4] Cohen E., Casida J.E. (1982). Properties and inhibition of insect integumental chitin synthase. Pestic. Biochem. Physiol..

[5] Sakuda S., Musumeci S., Paoletti M.G. (2009). The biochemical significance of allosamidins as chitinase inhibitors. Binomium Chitin-Chitinase: Resent Issues.

[6] Sakuda S., Isogai A., Matsumoto S., Suzuki A., Koseki K. (1986). Structure of allosamidin, a novel insect chitinase inhibitor, Produced by *Streptomyces* sp.. Tetrahedron Lett..

[7] Sakuda S., Isogai A., Matsumoto S., Suzuki A. (1987). Search for microbial insect growth regulators II. Allosamidin. A novel insect chitinase inhibitor. J. Antibiot..

[8] Sakuda S., Isogai A., Makita T., Matsumoto S., Koseki K., Kodama H., Suzuki A. (1987). Structures of allosamidins, Novel insect chitinase inhibitors, Produced by actinomycetes. Agric. Biol. Chem..

[9] Sakuda S., Isogai A., Matsumoto S., Suzuki A., Koseki K., Kodama H., Yamada Y. (1988). Absolute configuration of allosamizoline, an aminocyclitol derivative of the chitinase inhibitor allosamidin. Agric. Biol. Chem..

[10] Zhou Z.Y., Sakuda S., Yamada Y. (1992). Biosynthetic studies on the chitinase inhibitor, allosamidin. Origin of the carbon and nitrogen atoms. J. Chem. Soc. Perkin Trans. I.

[11] Zhou Z.Y., Sakuda S., Kinoshita M., Yamada Y. (1993). Biosynthetic studies of allosamidin 2. Isolation of didemethylallosamidin, and conversion experiments of ^14^C-labeled demethylallosamidin, didemethylallosamidin and their related compounds. J. Antibiot..

[12] Sakuda S., Sugiyama Y., Zhou Z.Y., Takao H., Ikeda H., Kakinuma K., Yamada Y., Nagasawa H. (2001). Biosynthetic studies on the cyclopentane ring formation of allosamizoline, an aminocyclitol component of the chitinase inhibitor allosamidin. J. Org. Chem..

[13] Nishimoto Y., Sakuda S., Takayama S., Yamada Y. (1991). Isolation and characterization of new allosamidins. J. Antibiot..

[14] Berecibar A., Grandjean C., Siriwardena A. (1999). Synthesis and biological activity of natural aminocyclopentitol glycosidase inhibitors: Mannostatins, Trehazoline, Allosamidins, And their analogues. Chem. Rev..

[15] Anderses O.A., Dixon M.J., Eggleston I.M., van Aalten D.M.F. (1005). Natural product family 18 chitinase inhibitors. Nat. Prod. Rep..

[16] Gangliang H. (2012). Recent progress on synthesis and activities of allosamidin and its analogues. Med. Chem..

[17] Sakuda S., Isogai A., Suzuki A., Yamada Y. (1993). Chemistry and biochemistry of the chitinase inhibitors, allosamidins. Actinomycetologica.

[18] Henrissat B.A. (1991). Classification of glycosyl hydrolases based on amino acid sequence similarities. Biochem. J..

[19] Spindler K.D., Spindler-Barth M., Jolles P., Muzzarelli R.A.A. (1999). Inhibitor of chitinases. Chitin and Chitinases.

[20] Tews I., van Scheltinga A.C.T., Perrakis A., Wilson K.S., Dijkstra B.W. (1997). Substrate-assisted catalysis unifies two families of chitinolytic enzymes. J. Am. Chem. Soc..

[21] van Scheltinga A.C.T., Armand S., Kalk K.H., Isogai A., Henrissat B., Dijkstra B.W. (1995). Stereochemistry of chitin hydrolysis by a plant chitinase/lysozyme and X-ray structure of a complex with allosamidin: Evidence for substrate assisted catalysis. Biochemstry.

[22] Brameld K.A., Shrader W.D., Imperiali B., Gddard W.A. (1998). Substrate assistance in the mechanism of family 18 chitinases: Theoretical studies of potential intermediates and inhibitors. J. Mol. Biol..

[23] Germer A., Klod S., Peter M.G., Kleinpeter E. (2002). NMR spectroscopic and theoretical study of the complexation of the inhibitor allosamidin in the binding pocket of the plant chitinase hevamine. Mol. Model..

[24] Papanikolau Y., Tavlas G., Vorgias C.E., Petratos K. (2003). De novo purification scheme and crystallization conditions yield high-resolution structures of chitinase A and its complex with the inhibitor allosamidin. Acta Cryst..

[25] van Aalten D.M.F., Komander D., Synstad B., Gaseidnes S., Peter M.G., Eijsink V.G.H. (2001). Structural insights into the catalytic mechanism of a family 18 exo-chitinase. Proc. Natl. Acad. Sci. USA.

[26] Bortone K., Monzingo A.F., Ernst S., Robertus J.D. (2002). The structure of an allosamidin complex with the *Coccidioides immitis* chtinase defines a role for a second acid residue in substrate-assisted mechanism. J. Mol. Biol..

[27] Rao F.V., Houston D.R., Boot R.G., Aerts J.M.F., Sakuda S., van Aalten M.F. (2003). Crystal structures of allosamidin derivatives in complex with human macrophage chitinase. J. Biol. Chem..

[28] Zhao Y.S., Zheng Q.C., Zhang H.X., Chu H.Y., Sun C.C. (2009). Analysis of a three-dimensional structure of human acidic mammalian chitinase obtained by homology modeling and ligand binding studies. J. Mol. Model..

[29] Cederkvist F.H., Saua S.F., Karlsen V., Sakuda S., Eijsink V.G.H., Sorlie M. (2007). Thermodynamic analysis of allosamidin binding to a family 18 chitinase. Biochemistry.

[30] Zakariassen H., Klemetsen L., Sakuda S., Vaaje-Kolstad G., Varum KM., Sorlie M., Eijsink V.G.H. (2010). Effect of enzyme processivity on the efficacy of a competitive chitinase inhibitor. Carbohydr. Polym..

[31] Baban J., Fjeld S., Sakuda S., Eijsink V.G.H., Sorlie M. (2010). The roles of three *Serratia marcescens* chitinases in chitin conversion are reflected in different thermodynamic signatures of allosamidin binding. J. Phys. Chem. B.

[32] Blattner R., Gerard P.J., Spindler-Barth M. (1997). Synthesis and biological activity of allosamidin and allosamidin analogues. Pestic. Sci..

[33] Somers P.J.B., Yao R.C., Doolin L.E., McGowan M.J., Fukuda D.S., Mynderse J.S. (1987). Methods for detection and quantitation of chitinase inhibitors in fermentation broths; isolation and insect life cycle effect of A82516. J. Antibiot..

[34] Filho B.P.D., Lemos F.J.A., Secundino N.F.C., Pascoa V., Pereira S.T., Pimenta P.F.P. (2002). Presence of chitinase and beta-*N*-acetyl glucosaminidase in the *Aedes aegypti* a chitinolytic system involving peritrophic matrix formation and degradation. Insect Biochem. Mol. Biol..

[35] Sakuda S., Nishimoto Y., Ohi M., Watanabe M., Takayama S., Isogai A., Yamada Y. (1990). Effects of demethylallosamidin, a potent yeast chitinase inhibitor, on the cell division of yeast. Agric. Biol. Chem..

[36] Yamanaka S., Tsuyoshi N., Kikuchi R., Takayama S., Sakuda S., Yamada Y. (1994). Effect of demethylallosamidin, a chitinase inhibitor, on morphology of fungus *Geotrichum candidum*. J. Gen. Appl. Microbiol..

[37] Adams D.J. (2004). Fungal cell wall chitinases and glucanases. Microbiology.

[38] Dickinson K., Keer V., Hitchcock C.A., Adams D.J. (1989). Chtinase activity from *Candida albicans* and its inhibition by allosamidin. J. Gen. Microbiol..

[39] Yamazaki H., Yamazaki D., Takaya N., Takagi M., Ohta M., Horiuchi H. (2007). A chitinase gene, chiB, involved in the autolysis process of *Aspergillus nidulans*. Curr. Genet..

[40] Sami L., Pusztahelyi T., Emri T., Varecza Z., Fekete A., Grallert A., Karanyi Z., Kiss L., Pocsi I. (2001). Autolysis and aging of *Penicillium chrysogenum* cultures under carbon starvation: Chitinase production and antifungal effect of allosamidin. J. Gen. Appl. Microbiol..

[41] Villagomez-Castro J.C., Calvo-Mendez C., Lopez-Romero E. (1992). Chtinase activity in encysting *Entamoeba invadens* and its inhibition by allosamidin. Mol. Biochem. Parasitol..

[42] Shahabuddin M., Toyoshima T., Aikawa M., Kaslow D.C. (1993). Transmission-blocking activity of a chitinase inhibitor and activation of malarial parasite chitinase by mosquito protease. Proc. Natl. Acad. Sci. USA.

[43] Vinetz J.M., Dave S.K., Specht C.A., Brameld K.A., Xu B., Hayward R., Fidock D.A. (1999). The chitinase PfCHT1 from the human malaria parasite *Plasmodium falciparum* lacks proenzyme and chitin-binding domains and displays unique substrate preferences. Proc. Natl. Acad. Sci. USA.

[44] Takeo S., Hisamori D., Matsuda S., Vinetz J., Sattabongkot J., Tsuboi T. (2009). Enzymatic characterization of the Plasmodium vivax chitinase, a potential malaria transmission-blocking target. Parasitol. Int..

[45] Wu Y., Egerton G., Underwood A.P., Sakuda S., Bianco A.E. (2001). Expression and secretion of a larval-specific chtinase (family 18 glycosyl hydrolase) by the infective stages of the parasitic nematode, *Onchocerca volvulus*. J. Biol. Chem..

[46] Takenaka Y., Nakano S., Tamoi M., Sakuda S., Fukamizo T. (2009). Chitinase gene expression in response to environmental stresses in *Arabidopsis thaliana*: Chitinase inhibitor allosamidin enhances the stress. Biosci. Biotechnol. Biochem..

[47] Sampson M.N., Gooday G.W. (1998). Involvement of chitinases of *Bacillus thuringiensis* during pathogenesis in insects. Microbiology.

[48] Boer W.D., Gunnewiek P.J.A.K., Kowalchuk G.A., van Veen J.A. (2001). Growth of chitinolytic dune soil β-subclass *Proteobacteria* in response to invading fungal hyphae. Appl. Environ. Microbiol..

[49] Suzuki S., Nakanishi E., Ohira T., Kawachi R., Nagasawa H., Sakuda S. (2006). Chitinase inhibitor allosamidin is a signal molecule for chitinase production in its producing *Streptomyces*. I. Analysis of the chitinase whose production is promoted by allosamidin and growth accelerating activity of allosamidin. J. Antibiot..

[50] Suzuki S., Nakanishi E., Ohira T., Kawachi R., Ohnishi Y., Horinouchi S., Nagasawa H., Sakuda S. (2006). Chitinase inhibitor allosamidin is a signal molecule for chitinase production in its producing *Streptomyces*. II. Mechanism for regulation of chitinase production by allosamidin through a two-component regulatory system. J. Antibiot..

[51] Suzuki S., Nakanishi E., Furihata K., Miyamoto K., Tsujibo H., Watanabe T., Ohnishi Y., Horinouchi S., Nagasawa H., Sakuda S. (2008). Chtinase inhibitor allosamidin promotes chitinase production of *Streptomyces* generally. Int. J. Biol. Macromol..

[52] Zhu Z., Zheng T., Homer R.J., Kim Y.K., Chen N.Y., Cohn L., Hamid Q., Elias J.A. (2004). Acidic mammalian chitinase in asthmatic Th2 inflammation and IL-13 pathway activation. Science.

[53] Bucolo C., Musumeci M., Maltese A., Drago F., Musumeci S. (2008). Effect of chitinase inhibitors on endotoxin-induced uveitis (EIU) in rabbits. Pharmacol. Res..

[54] Kitamoto S., Egashira K., Ichiki T., Han X., McCurdy S., Sakuda S., Sunagawa K., Boisvert W.A. (2013). Chitinase inhibition promotes atherosclerosis in hyperlipidemic mice. Am. J. Pathol..

[55] Lingappa Y., Lockwood J.L. (1961). A chitin medium for isolation, growth and maintenance of actinomycetes. Nature.

[56] Bentley S.D., Chater K.F., Cerdeño-Tárraga A.M., Challis G.L., Thomson N.R., James K.D., Harris D.E., Quail M.A., Kieser H., Harper D. (2002). Complete genome sequence of the model actinomycete *Streptomyces coelicolor* A3(2). Nature.

[57] Ni X., Westpheling J. (1997). Direct repeat sequences in the *Streptomyces* chitinase-63 promoter direct both glucose repression and chitin induction. Proc. Natl. Acad. Sci. USA.

[58] Saito A., Ishizaka M., Francisco P.B., Fujii T., Miyashita K. (2000). Transcriptional co-regulation of five chitinase genes scattered on the *Streptomyces coelicolor* A3(2) chromosome. Microbiology.

[59] Saito A., Schrempf H. (2004). Mutational analysis of the binding affinity and transport activity for *N*-acetylglucosamine of the novel ABC transporter Ngc in the chitin-degrader *Streptomyces olivaceoviridis*. Mol. Genet. Gen..

[60] Wang Q., Zhou Z.Y., Sakuda S., Yamada Y. (1993). Purification of allosamidin-sensitive and -insensitive chitinases produced by allosamidin-producing *Streptomyces*. Biosci. Biotech. Biochem..

[61] Ohno T., Armand S., Hara T., Nikaidou N., Henrissat B., Mitsutomi M., Watanabe T. (1996). A modular family 19 chitinase found in the prokaryotic organism *Streptomyces griseus* HUT 6037. J. Bacteriol..

[62] Matsuura H., Okamoto S., Anamnart S., Wang Q., Zhou Z.Y., Nihira T., Yamada Y., Kuzuyama T., Seto H., Nakayama J., Suzuki A., Nagasawa H., Sakuda S. (2003). Nucleotide sequences of genes encoding allosamidin-sensitive and -insensitive chitinases produced by allosamidin-producing *Streptomyces*. Biosci. Biotech. Biochem..

[63] Nakanishi E., Okamoto S., Matsuura H., Nagasawa H., Sakuda S. (2001). Allosamidin, a chitinase inhibitor produced by *Streptomyces*, acts as an inducer of chitinase production in its producing strain. Proc. Japan Academy Ser. B.

[64] Wills-Karp M., Luyimbazi J., Xu X., Schofield B., Neben T.Y., Karp C.L., Donaldson D.D. (1998). Interleukin-13: Central mediator of allergic asthma. Science.

[65] Boot R.G., Blommaart E.F.C., Swart E., Ghauharali-van der Vlugt K., Bijl N., Moe C., Place A., Aerts M.F. (2001). Identification of a novel acidic mammalian chitinase distinct from chitotriosodase. J. Biol. Chem..

[66] Boot R.G., Bussink A.P., Verhoek M., de Boer P.A.J., Moorman A.F.M., Aerts J.M.F.G. (2005). Marked diffrences in tissue-specific expression of chitinases in mouse and man. J. Histochem. Cytochem..

[67] Homer R.J., Zhu Z., Cohn L., Lee C.G., White W.I., Chen S., Elias J.A. (2006). Differential expression of chitinases identify subsets of murine airway epithelial cells in allergic inflammation. Am. J. Physiol. Lung Cell Mol. Physiol..

[68] Matsumoto T., Inoue H., Sato Y., Kita Y., Nakano T., Noda N., Eguchi-Tsuda M., Moriwaki A., Kan-o K., Matsumoto K., Shimizu T., Nagasawa H., Sakuda S., Nakanishi Y. (2009). demethylallosamidin, a chitinase inhibitor, suppresses airway inflammation and hyperresponsiveness. Biochem. Biophys. Res. Commun..

[69] Karasuda S., Yamamoto K., Kono M., Sakuda S., Koga D. (2004). Kinetics analysis of a chitinase from red sea bream, *Pagrus major*. Biosci. Biotech. Biochem..

[70] Sato Y., Suzuki S., Muraoka S., Kikuchi N., Noda N., Matsumoto T., Inoue H., Nagasawa H., Sakuda S. (2011). Preparation of allosamidin and demethylallosamidin photoaffinity probes and analysis of allosamidin-binding proteins in asthmatic mice. Bioorg. Med. Chem..

[71] Ober C., Chupp G.L. (2009). The chitinase and chitinase-like proteins: A review of genetic and functional stidies in asthma and immune-mediated diseases. Curr. Opin. Allergy Clin. Immunol..

[72] Sutherland T.E., Maizels R.M., Allen J.E. (2009). Chitinases and chitinase-like proteins: Potential therapeutic targets for the treatment of T-helper type 2 allergies. Clin. Experi. Allergy.

[73] Owashi M., Arita H., Hayai N. (2000). Identification of a novel eosinophil chemotactic cytokine (ECF-L) as a chitinase family protein. J. Biol. Chem..

[74] Houston D.R., Recklies A.D., Krupa J.C., van Aalten D.M.F. (2003). Structure and ligand-induced conformational change of the 39-kDa glycoprotein from human articular chondrocytes. J. Biol. Chem..

[75] Schimpl M., Rush C.L., Betou M., Eggleston I.M., Recklies A.D., van Aalten D.M.F. (2012). Human YKL-39 is apseudo-chitinase with retained chitooligosaccharide-binding properties. Biochem. J..

[76] Lee C.G., Elias J.A. (2010). Role of breast regression protein-39/YKL-40 in asthma and allergic responses. Allergy Asthma Immunol. Res..

[77] Iwashita H., Morita S., Sagiya Y., Nakanishi A. (2006). Role of eosinophil chemotactic factor by T lymphocytes on airway hyperresponsiveness in a murine model of allergic asthma. Am. J. Respir. Cell Mol. Biol..

[78] Lee C.G., Hartl D., Lee G.R., Koller B., Matsuura H., Da Silva C.A., Sohn M.H., Cohn L., Homer R.J., Kozhich A.A. (2009). Role of breast regression protein 39 (BRP-39)/chitinase 3-like-1 in Th2 and IL-13-induced tissue responses and apoptosis. J. Exp. Med..

[79] Cai Y., Kumar R.K., Zhou J., Foster P.S., Webb D.C. (2009). Ym1/2 promotes Th2 cytokine expression by inhibiting 12/15(*S*)-lipoxygenase: Identification of a novel pathway for regulating allergic inflammation. J. Immunol..

